# Vascular Cell Adhesion Molecule 1 and E-Selectin as Potential Cardiovascular Risk Biomarkers in Psoriasis

**DOI:** 10.3390/ijms26020792

**Published:** 2025-01-18

**Authors:** Natalia Joanna Machoń, Natalia Zdanowska, Paulina Klimek-Trojan, Agnieszka Owczarczyk-Saczonek

**Affiliations:** 1 School of Medicine, Collegium Medicum, University of Warmia and Mazury, 10-082 Olsztyn, Poland; mnatalia000@gmail.com; 2 Department of Dermatology, Sexually Transmitted Diseases and Clinical Immunology, Collegium Medicum, University of Warmia and Mazury, Al. Wojska Polskiego 30, 10-229 Olsztyn, Poland

**Keywords:** VCAM-1, E-selectin, psoriasis, cardiovascular risk, biomarkers

## Abstract

Vascular cell adhesion molecule-1 (VCAM-1) and E-selectin are involved in different inflammatory diseases and may be potential cardiovascular risk biomarkers in psoriasis. They play an important role in regulating the recruitment and adhesion to endothelial cells during inflammation, affecting various conditions like vasculitis, atherosclerosis, and cardiovascular diseases. Positive outcomes have been observed when using Tumor Necrosis Factor Alpha (TNF-α) inhibitors and biological therapies that target selectins to control the functioning of endothelial cells and reduce inflammation in psoriasis and related conditions. Moreover, the effects of systemic treatments and ultraviolet B (UVB) phototherapy on VCAM-1 and E-selectin levels in psoriasis patients highlights the potential to impact the severity of psoriasis and activation of endothelial cells. In addition, various factors such as age, sex, metabolic syndrome, hyperglycemia, migraines, and tobacco smoking have been found to affect levels of VCAM-1 and E-selectin. This sheds light on understanding the complex relationship between endothelial activation and the development of diseases. Studies show the potential of using the levels of VCAM-1 and E-selectin as indicators of systemic treatment effectiveness and the progression of the disease. In summary, this review highlights the importance of VCAM-1 and E-selectin as potential biomarkers for assessing inflammation, disease severity and cardiovascular risk in individuals with psoriasis. The shared mechanisms of psoriasis and atherosclerosis, along with the effect of treatments on endothelial activation markers, provide significant insights for further research and approaches to manage inflammatory diseases in the future.

## 1. Introduction

Psoriasis is a chronic inflammatory skin disease with a prevalence ranging from 0.1 to 8%. The disease manifests with both typical skin and systemic lesions, as confirmed by epidemiological data. More than 80% of psoriasis cases are plaque psoriasis, which is its most common form. The skin lesions are also accompanied by subjective complaints such as itching, burning or soreness of the skin. Among the comorbidities associated with psoriasis, it is important to note those also associated with systemic inflammation, such as psoriatic arthritis, cardiovascular disease, metabolic syndrome, obesity, inflammatory bowel disease, and psychiatric disorders [1].

The multifactorial etiology of the disease includes both extrinsic and intrinsic factors. Genes involved in antigen presentation (human leukocyte antigen (HLA)-Cw6), cytokine signaling (interleukin (IL)-12B, IL23R), interferon signaling and nuclear factor kappa B (NF-κB) signaling (tumor necrosis factor, alpha-induced protein 3 (TNFAIP3), nuclear factor-kappa-B-inhibitor alpha (NFKBIA), nuclear factor-kappa-B inhibitor zeta (NFKBIZ), TNFAIP3-interacting protein 1 (TNIP1) and reticuloendotheliosis viral oncogene homolog A (RELA)) are among the genetic risk factors. In the pathogenesis of psoriasis, inflammatory pathways in both innate and acquired immunity mechanisms are activated. This leads to uncontrolled keratinocyte (KC) proliferation, acanthosis, neovascularization, and an influx of immune cells into the skin [1].

Vascular cell adhesion molecule 1 (VCAM-1 (CD106)) is a 90 kDa glycoprotein that is expressed on endothelial cells, but also in other cell types (dendritic cells, bone marrow stromal cells, macrophages, respiratory epithelia, myoblasts, Kupfer cells or Sertoli cells). The structure of VCAM-1 comprises a cytoplasmic tail, a trans membrane region and six-seven immunoglobulin-like domains forming the trans membrane region. The first VCAM-1 isoform contains seven immunoglobulin-like domains, while the second isoform lacks one domain. Participation in lymphocyte adhesion and migration is initiated by the expression of VCAM-1 on endothelial cells. Infiltration of leukocytes into inflamed tissues is possible through an alteration of the actin cytoskeleton and incompressible junctions within the endothelium. Under inflammatory conditions, secreted TNF alpha influences the increased expression of VCAM-1. Signalling pathways initiated by integrin α4β1 binding lead to calcium activation, NADPH oxidase 2 (NOX2), ROS production, matrix metalloproteinase (MMP). This ensures precise recruitment and activation of leukocytes.

VCAM-1 plays a significant role in maintaining homeostasis and is involved in various pathological conditions, including cancer, atherosclerosis, atrial fibrillation, myocardial infarction, stroke, asthma, obesity, and kidney diseases [2,3].

Selectin E (Endothelial Leukocyte Adhesion Molecule-1, ELAM1, CD62E, LECAM-2) consists of five different structural domains. Ligand binding is enabled by a pectin-like domain at the N-terminus. Others include an epidermal growth factor (EGF)-like domain, complement regulator-like domains, a trans-membrane segment and a cytoplasmic tail at the C-terminus [4]. E-selectin enables circulating leukocytes to adhere to the vascular endothelium, especially during inflammatory processes [4]. Its expression starts a few hours after the inflammation-initiating signal and is internalised or removed from the cell surface within about 24 hours [5].

The interaction of E and L selectin enables slow leukocyte rolling, adhesion and diapedesis. The binding of E-selectin to sLex on neutrophils induces a shift of integrin β2 (LFA-1) from a low- to intermediate-affinity state. is responsible for the activation of the inflamasome, leading to the release of myeloid-related protein 8/14 (MRP8/14, also known as S100A8/9 or calprotectin), which interacts with toll-like receptor 4 (TLR4). The increased affinity of LFA-1 for intercellular adhesion molecule-1 (ICAM-1) enables the initiation of strong adhesion after the slow rolling stage and subsequent migration into inflamed tissues [5].

The soluble form of E-selectin (sE-selectin) is produced by cleaving the membrane-bound molecule. Elevated levels of sE-selectin in the circulation serve as markers of endothelial cell activation in response to inflammation [4]. Under physiological conditions, E-selectin remains inactive in endothelial cells, but in response to oxidative stress or pro-inflammatory molecules (IL-1), bacterial lipopolysaccharide (LPS), TNF or viral infections [4]. The level of sE-selectin correlates with the presence and severity of disease [5]. Increased sE-selectin levels have been described in atopic dermatitis, psoriasis, inflammatory bowel disease, sarcoidosis, systemic scleroderma and lupus. Furthermore, elevated sE-selectin levels correlate with disease activity and prognosis in rheumatoid arthritis [5]. On the other hand, the information regarding adhesion molecules is inconsistent, with conflicting findings even in similar conditions like cardiovascular disease [6].

In this review, we aim to introduce the theory that these adhesion molecules may serve as potential cardiovascular risk biomarkers in psoriasis.

## 2. The Correlation Between Psoriasis and E-Selectin and VCAM-1

Memory T cells are involved in both the early and ongoing stages of psoriatic plaques formation. The molecules E-selectin and VCAM-1 found on the endothelium are crucial for the initial movement of memory T cells into psoriatic lesions [7].

Early-stage vascular changes in psoriasis contribute to increased blood flow through the skin. In addition to morphological changes, the papillary skin vessels in psoriasis show high levels of expression of inflammatory adhesion molecules, such as E-selectin, ICAM-1 and VCAM-1, which facilitate leukocyte adhesion to endothelial cells, thereby promoting the development of an inflammatory response [8].

**Figure 1:**
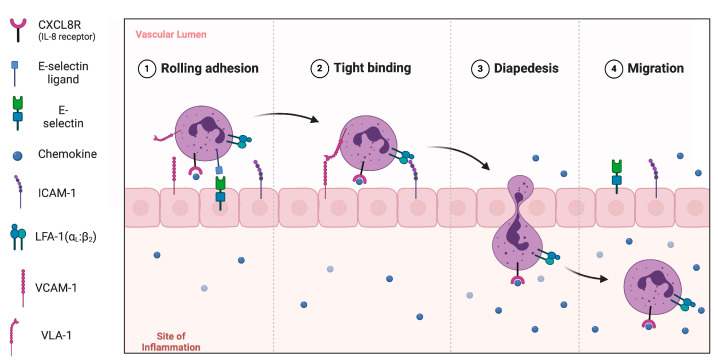
Model of leukocyte adhesion cascade involved in immune cell infiltration. Process involving rolling, adhesion, tight binding, diapedesis, and migration moves neutrophils towards the inflammation site. This cascade is facilitated by molecules interacting with each other. Abbreviations: LFA-1: Lymphocyte function-associated antigen 1, VLA-1: Very Late Antigen-1, ICAM-1: Intercellular adhesion molecule 1, VCAM-1: Vascular cell adhesion molecule-1, CXCL8R: interleukin-8 receptor. Own elaboration based on: [9,10] Figure was made using BioRender software.

When the skin is inflamed, neutrophils reach this site, where they are slowed down on activated endothelial cells in the process of rolling adhesion and tight binding. These processes are mediated by certain adhesion molecules and their receptors like: LFA-1 (Lymphocyte function-associated antigen 1), VLA-1 (Very Late Antigen-1), ICAM-1, VCAM-1, CXCL8R (interleukin-8 receptor). Neutrophils then flatten and eventually migrate through the endothelial lining into the surrounding connective tissue. These processes are demonstrated in Figure 1 [9,10].

An important role in the pathogenesis of psoriasis and other inflammatory diseases is provided by endothelial dysfunction characterized by, among other features, increased oxidative stress, leukocyte adhesion, and excessive permeability. Endothelial cells act as both sensors responding to stimuli in the psoriasis microenvironment and effectors [11].

Garshick et al. carried out research on the vascular endothelial health and changes in certain biological pathways in individuals with psoriasis compared to a control group. They found that people with psoriasis showed the most significant differences in the inflammasome pathway. Profiling of brachial vein endothelial cells that were collected showed an increase in inflammatory genes (such as IL-1β, CXC motif chemokine 10 (CXCL10), VCAM-1, IL-8, CXC motif chemokine ligand 1 (CXCL1), Lymphotoxin beta, ICAM-1, cyclooxygenase-2 (COX-2), and chemokine CC motif ligand 3 (CCL3)) in individuals with psoriasis compared to the control group. The correlation between inflammasome signaling and the severity of psoriasis, as well as the levels of circulating interleukin 6 and pro-inflammatory endothelial transcripts, was observed. These discoveries assist in providing a clearer understanding of why there is an increased likelihood of developing cardiovascular disease in individuals with psoriasis [12].

**Figure 2:**
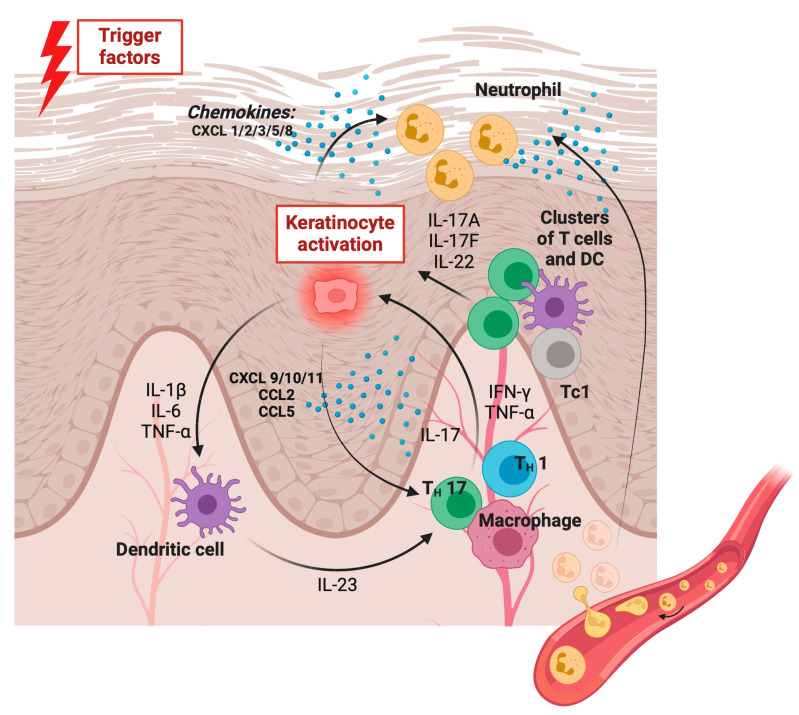
The cascade of neutrophil recruitment to inflamed skin is mediated by a finely tuned sequence of adhesive interactions. Neutrophils circulating in the bloodstream reach the inflamed skin. Abbreviations: CCL—chemokine C-C motif ligand; CCR—chemokine C-C motif receptor; CXCL—C-X-C motif chemokine ligand; CXCR—C-X-C motif chemokine receptor; DC—dendritic cell; Th1—T helper 1 lymphocyte; Th17—T helper 17 lymphocyte; Th2—T helper 2 lymphocyte; IL—interleukin; IFN-γ—interferon gamma; TNF-α—tumor necrosis factor alpha. Own elaboration based on [9,15] Figure was made using BioRender software.

Nowadays, psoriasis is acknowledged as a distinct risk factor for developing coronary heart disease and experiencing cardiovascular mortalities [13]. It shares several similarities with atherosclerosis in terms of immune system activation, cytokine communication, and the process of angiogenesis. Both conditions share common features, including the presence of activated T cells and macrophages associated with atherosclerosis in the impacted region. This infiltration is facilitated by the interaction between adhesion molecules and selectins, which helps the migration of these cells from the blood vessels [14]. The recruitment of neutrophils to inflamed skin in psoriasis occurs through a finely tuned sequence of adhesion interactions demonstrated in Figure 2 [9].

Chemokines play a role in the recruitment and activation of T cells, macrophages, and neutrophils in psoriatic inflammation. Among them can be distinguished those with inflammatory (CXCL1, 2, 3, 5, 6, 7 and 8), homeostatic (CXCL12 and 13) and both homeostatic and pro-inflammatory (CXCL9, 10, 11 and 16) functions. In the regional lymph node, a differentiation of naive T lymphocytes into Th1 and Th2 lymphocytes occurs, which is induced by dendritic cells. These cells release IL-23, while lymphocytes Th17 produce IL-17A, IL-17F, and IL-22 and lymphocytes Th1 produce interferon (IFN)-γ and TNF-α. Clusters of T cells and dendritic cells gather around blood vessels due to chemokines released by macrophages, such as CCL19. Meanwhile, CXCL1 and IL-8 (CXCL8) play a role in drawing neutrophils to the epidermis [9,15].

In Teixeira’s study, it was observed that individuals with psoriasis had higher levels of VCAM-1 and E-selectin in their plasma, in comparison to the control group [16].

Measurement of soluble E-selectin levels in the serum can be considered a dependable indicator of the severity of psoriatic disease in patients. Bonifati et al. observed that psoriatic patients had noticeably higher levels of sE-selectin in their blood compared to healthy individuals. Moreover, a significant correlation was identified between the levels of sE-selectin and PASI (psoriasis area and severity index) scores [17].

Szepietowski et al. proved that the levels of sE-selectin in the bloodstream are directly related to the severity of psoriasis, and therefore, can be used as an indicator of how active the disease is in patients. The study found that psoriatic patients had noticeably higher levels of sE-selectin in their blood compared to healthy individuals. Additionally, a strong correlation was shown between sE-selectin values and PASI scores. There was no correlation observed between the duration of psoriasis and the levels of sE-selectin. The serum levels of soluble E-selectin decreased significantly following the treatment of psoriasis [18]. Carducci et al. discovered notable connections between the PASI scores, corneometry of affected skin, and serum levels of E-selectin. Patients were examined to measure the moisture levels in both affected and unaffected areas of their skin, as well as their levels of E-selectin in the blood. It had been previously observed that psoriasis patients had higher levels of E-selectin before and after treatment. At the beginning, there was a strong association between the corneometry and E-selectin measurements, and the levels of PASI, infiltration, and desquamation scores. After therapy, the improvement of the lesion was associated with reduced PASI scores and E-selectin levels, as well as higher corneometric levels [19].

The findings of D’Auria et al. indicated that individuals with psoriasis and bullous dermatoses had higher levels of sE-selectin in their serum compared to healthy individuals. The difference in levels between the two groups was statistically significant. In addition, a significant connection was observed between the levels of serum and the severity of the disease, as indicated by the PASI score in psoriasis patients. Similar correlations were observed in bullous dermatoses, where serum levels were significantly correlated with the number of visible lesions, such as blisters and erosions. Following the therapy, there was a notable reduction in marker levels [20].

Czech et al. demonstrated that the levels of soluble E-selectin were markedly increased in the blood of individuals with atopic dermatitis and psoriasis when compared to those without these conditions. There was a notable reduction in soluble E-selectin levels in patients treated for atopic dermatitis, leading to clinical improvement. However, this improvement was not observed in patients with psoriasis [21].

Sabovic et al. conducted a study in which they attempted to assess endothelial function by measuring arterial stiffness and circulating markers of endothelial activation in patients with plaque psoriasis undergoing successful treatment defined as achieving a PASI of 90 and in a group of healthy volunteers. Patients were treated with topical, classical drugs (methotrexate) and biologics (adalimumab, secukinumab, guselkumab), and the circulating markers assessed were ICAM-1, VCAM-1, E- and P-selectin, GDF-15, and TRAIL. There was an increase in circulating E-selectin levels in patients treated with secukinumab; other than that, there were no significant differences in arterial stiffness and levels of other biomarkers compared to the control group [22].

In recent times, there has been a suggestion that the level of Circulating Endothelial Cells (CECs) could serve as a new indicator of vascular injury. The increased levels of CECs observed in a particular set of psoriatic patients may indicate damage to the endothelium. The decrease in CEC count following treatment with Etanercept suggests that a successful treatment for psoriasis could potentially enhance the functioning of the endothelial cells. The study showed a significant difference in the CEC level between psoriatic patients and a control group. The count had an inverse correlation with sE-selectin levels. After undergoing therapy for a period of 6 months, patients showed a notable reduction in CEC levels and in their PASI score [23].

Given the significant amount of evidence that supports the important role of E-selectin in the movement of leukocytes observed during the inflammatory process caused by psoriasis and atherosclerosis, these markers of endothelial cell activation may have the potential to be utilized to evaluate inflammation, disease severity, and cardiovascular disease caused by atherosclerosis in patients with psoriasis [24].

The epidemiological data provides evidence for the link between psoriasis and negative cardiovascular outcomes. Endothelial dysfunction is believed to play a role in both psoriasis and atherosclerosis, suggesting a common disease mechanism between the two conditions [25].

## 3. Effect of Systemic Treatment or UVB Phototherapy in Psoriasis on VCAM-1 and E-Selectin Levels

### 3.1. Phototherapy

Increased levels of adhesion molecules ICAM-1 and VCAM-1 in both affected and unaffected skin in individuals with psoriasis indicate the importance of these molecules in the development of the disease. Studies have shown that UVA and UVB treatments decrease the presence of these molecules. The findings reported by Cabirjan et al. reveal higher levels of ICAM-1 molecules in keratinocytes, in the perivascular infiltrate (specifically lymphocytes), and in endothelial cells. The level of VCAM-1 molecules was also elevated, but not as strongly as ICAM-1. After undergoing the therapy, there was a notable reduction in the presence of adhesion molecules, which subsequently resulted in significant improvement in the disease’s condition [26,27].

The observations of Cai et al. indicate that UVB treatment disrupts the ability of endothelial cells to stick together and selectively controls the expression of adhesive molecules in these cells [28].

The research conducted by Long et al. investigated how narrow-band ultraviolet B (NB-UVB) phototherapy impacted the levels of certain soluble cell adhesion molecules (sE-selectin, sP-selectin, sL-selectin, and soluble intercellular adhesion molecule-1 (sICAM-1)) in patients with psoriasis vulgaris. The effectiveness of NB-UVB phototherapy was confirmed as the treatment led to a significant reduction in PASI scores. The levels of sE-selectin in the serum also significantly decreased following the treatment. Additionally, the levels of sICAM-1 exhibited a strong correlation with both the PASI score and the levels of sE-selectin. The effectiveness of NB-UVB phototherapy in treating psoriatic lesions may be due to a reduction in the amount of E-selectin in serum [29].

### 3.2. Fumarates

In addition to its ability to inhibit the growth of keratinocytes, dimethylfumarate, which is used to treat psoriasis, may also have the ability to inhibit the expression of adhesion molecules induced by cytokines. Vandermeeren et al. showed that the inhibition of VCAM-1 and E-selectin expression was observed when using dimethylfumarate. There was no impact observed from monoethylfumarate and fumaric acid. This information supports the findings of previous studies on the impact of DMF on the levels of thiol molecules within cells and its ability to inhibit the activation of NF-kappa B [30].

Wallbrecht et al. demonstrated that the use of dimethylfumarate for treating psoriasis led to a significant decrease in the expression of E-selectin, ICAM-1, and VCAM-1 induced by TNFα on two distinct groups of endothelial cells, with the reduction becoming stronger as the concentration of dimethylfumarate increased. These findings indicate that endothelial cells experience significant functional alterations in response to dimethylfumarate, along with its already established effects on leukocytes. These alterations are accompanied by significantly impaired dynamic interactions with lymphocytes, which are the crucial initial process of the recruitment of white blood cells to inflamed tissues in psoriasis and other inflammatory disorders associated with TNF [31].

### 3.3. Methotrexate

The complete understanding of how methotrexate (MTX) works in treating psoriasis is still unknown. Torres-Alvares et al. conducted an immunohistochemical examination of the levels of T-cell characteristics and cell adhesion/activation molecules in skin samples obtained from individuals with psoriasis who received a consistent amount of MTX (12.5 mg/week). Before treatment, skin samples displayed a significant presence of inflammation, ranging from moderate to severe. The inflammation was primarily caused by T lymphocytes with a helper/inducer phenotype characterized by the CD4 marker. Most of these cells showed the presence of ICAM-1 and VCAM-1. Staining for E-selectin and VCAM-1 was observed in the blood vessels, while ICAM-1 staining was detected in the keratinocytes. The number of cells that had infiltrated decreased after treatment, and there was also a decrease in the expression of cell adhesion molecules [32].

In another study conducted by Sigmundsdottir et al., it was noticed that the patient’s clinical condition improved when they restarted the MTX treatment. This improvement was linked to a decrease in cutaneous lymphocyte-associated antigen (CLA) expression by mononuclear cells in the blood, a decrease in endothelial E-selectin, and a roughly threefold reduction in the infiltration of mononuclear leukocytes into the affected skin. The authors reached the conclusion that MTX reduces the levels of CLA and E-selectin, which may be a significant reason behind the positive impact of MTX on psoriatic skin lesions [33].

### 3.4. Biological Treatment

Biological therapies have emerged as a recent advancement in the management of psoriasis and psoriatic arthritis (PsA). Out of all these treatments, the most effective ones for controlling inflammation in the skin and joints are infliximab and etanercept, which are known as TNF-alpha antagonists. TNF is believed to play a vital role in initiating the cascade of cytokines that leads to inflammation in these areas [34]. Psoriasis is linked to a dysfunction in the endothelium, resulting in impaired functioning of blood vessels. TNF-α inhibitors have demonstrated the capacity to enhance the vascular function in individuals with psoriasis [35].

Brezinski et al. conducted a systematic review that explored the impact of TNF inhibitors on the function of the endothelium in individuals with psoriasis and psoriatic arthritis. This review indicates that the ability of blood vessels to function properly is noticeably reduced in individuals with psoriasis and PsA when compared to the general population. Early studies indicate that TNF inhibitors might enhance the functioning of endothelial cells in people with psoriasis and PsA [25]. 

Rios-Navarro et al. examined how anti-TNF-α drugs (adalimumab, infliximab, and etanercept) affected the interactions between human leukocytes and endothelial cells in a flow chamber that reproduced in vivo conditions. The effects of different anti-TNF-α concentrations in a clinical setting were examined on the mobilization of white blood cells caused by various substances involved in inflammation and the development of atherosclerosis such as TNF-α, interleukin-1β, lymphotoxin-α, angiotensin-II, PAF, IL-12, and IL-23. Treatment with anti-TNF-α, whether administered prior to or after the initiation of inflammation caused by TNF-α, reduced the interactions between leukocytes and endothelial cells that were induced by these stimuli. The findings also suggested that adhesion molecules (ICAM-1, VCAM-1, and E-selectin) play a role in how anti-TNF-α affects the adhesion of white blood cells to endothelium [36].

Mastroianni et al. conducted a study, in which 20 patients diagnosed with PsA and having PASI scores ranging from 0.4 to 42.8 were administered infliximab treatment for a period of 30 to 42 weeks. At the 10-week mark, 18 out of 20 patients with psoriasis experienced more than a 50% improvement in their PASI score, while 14 out of 20 patients saw more than a 75% improvement. At week 12, there was a notable decrease in TNF-α. On the other hand, IL-6, VEGF, FGF, and E-selectin displayed notable reductions following initial infliximab infusions. There was no correlation observed between PASI and TNF-α levels in the serum [34].

Cordiali-Fei et al. investigated the presence of E-selectin in the tissue and blood samples of psoriasis patients who were undergoing a six-week treatment with infliximab. The improvement in a clinical condition was linked to a notable reduction in E-selectin levels. These levels were previously observed to be released by affected tissue samples prior to treatment and continued to decrease after therapy. Furthermore, there were strong associations observed between the PASI measurements and the production of E-selectin [37].

Fernanda Gendre et al. demonstrated that patients with moderate-to-severe psoriasis exhibit endothelial activation, which is linked to the characteristics of metabolic syndrome. Treatment with adalimumab resulted in a decrease in sE-selectin levels, indicating that anti-TNF-α therapy has a positive impact on processes related to the progression of atherosclerosis in patients with psoriasis. The results support a recent study by Gkapalkiotis et al., which found that adalimumab (anti-TNF-α drug) therapy for psoriasis patients lowered the level of serum sE-selectin after three months of treatment [24,38].

Adalimumab may lead to a gradual and consistent decrease in blood vessel resistance in the nailfold area and the protein E-selectin in individuals with psoriasis. The nailfold vessel resistance index (NVRI) evaluates how well the small blood vessels in the nailfold area are functioning. Molina-Leva et al. conducted a study to evaluate the impact of inhibiting TNF-α using adalimumab on NVRI. In week 52, there was a decrease observed in NVRI and E-selectin levels [35].

However, even though in animal models of inflammatory diseases, the suppression of selectins and their ligands has demonstrated the effectiveness of this strategy in vivo, only a small number of drugs that target selectins have undergone testing in humans [39].

Bhushan et al. conducted a study that involved multiple centers and randomly assigned participants to receive either a humanized monoclonal antibody called CDP850 or a placebo. The aim was to assess the effectiveness and side effects of CDP850 in treating moderate to severe chronic plaque psoriasis. The results of the study indicated that while CDP850 was well tolerated and considered safe, it did not demonstrate any therapeutic benefits in treating chronic plaque psoriasis. This was evident as no significant reduction in the severity of the condition, as measured by the PASI score, was observed in either the group receiving CDP850 or the placebo group at the end of the study [40].

TNFα could potentially have a significant impact on activating vascular adhesion molecules in psoriatic lesions. Terajima et al. examined the expression of VCAM-1 and E-selectin by using immunohistochemistry in psoriatic skin (both involved and uninvolved areas) and compared it to their expression levels in normal skin. Dermal dendritic cells and fibroblasts showed significant staining for VCAM-1, while endothelial cells from affected skin exhibited notable staining for E-selectin [41].

IL-23 inhibitors, such as risankizumab and tildrakizumab, are also important drugs in the treatment of psoriasis. Their importance is particularly important in patients with current comorbidities such as obesity or in cases of severe psoriasis [42,43]. Brunasso et al. introduced a case series of patients with different concomitant diseases such like cardiovascular comorbidities, obesity, or with hard-to-treat areas of psoriasis plaques. Risankizumab rapidly improved symptoms in these patients [42]. Gargiulo et al. showed that a dose of tildrakizumab has a major influence on the effectiveness of the treatment. In patients with a body weight ≥ 90 kg, a high disease burden, 200 mg of tildrakizumab performed better [43].

## 4. The Prevalence of Comorbidities in Psoriasis

Psoriasis is an immune-mediated disease associated with an increased risk of concomitant disorders, in which there are common inflammatory mediators involved. Among the most frequent comorbidities are psoriatic arthritis, cardiovascular disease, metabolic syndrome comprising obesity and diabetes with insulin resistance, inflammatory bowel disease, psychiatric disorders (especially depression), multiple sclerosis, and malignancy. The shared pathogenic mechanism between psoriasis and different comorbidities increases the possibility of choosing an appropriate treatment that will be tailored to the individual patient [44].

Cardiovascular disease is much more (about 50%) prone to develop in psoriasis patients. The 10-year cardiovascular risk (CV) scales are used to assess CV risk. However, they do not take into account the increased risk that occurs in patients with psoriasis. In such patients, myocardial infarction can occur up to 5 years earlier than in people without this dermatitis. Because of the ongoing vascular inflammation that occurs in patients with psoriasis, the cardiovascular risk increases by 1% absolute for each year of the disease. It is also recommended that patients with psoriatic lesions occupying more than 10% of the body surface or patients treated with systemic or phototherapy should use a multiplier of 1.5 for a 10-year CV risk assessment [45].

In obese people, attention should also be paid to the psoriasis course. Adipokines (pro-inflammatory cytokines derived from adipose tissue) are elevated in these individuals and put neutrophils into a hyperactive state, which increases their innate immune response. This results in chronic inflammation in the body, which can affect the development of psoriasis [46].

## 5. The Role of VCAM-1 and E-Selectin in Atherosclerosis

Leukocyte migration into the tissues represents a key process in the pathogenesis of inflammatory diseases, and their rolling is the earliest step of their adhesion process in inflamed vessels [39]. Endothelial activation triggers the increase in E-selectin adhesion molecules, which facilitate the rolling of leukocytes along the vascular wall. This process is crucial in regulating inflammation in various conditions, such as atherosclerosis and heart failure [47]. A study by Eikendal et al. showed that in young adults, there is a positive correlation between the levels of E-selectin in the bloodstream and the thickness of the aortic wall and pulse wave velocity, as measured by cardiovascular magnetic resonance imaging. This suggests that these cell adhesion molecules may cause inflammatory changes in the arterial walls, leading to a higher risk of atherogenic diseases even at a young age [46].

The possibility of a connection between soluble adhesion molecules and atherosclerosis has been proposed, since atherosclerosis has similar features to a chronic inflammatory disorder. Soluble Vascular Cell Adhesion Molecule-1 (sVCAM-1) seems to exhibit higher specificity in identifying atherosclerosis when compared to other markers. The concentration of sVCAM-1 in the serum seems to be associated with the severity of atherosclerosis and could potentially be used to identify early stages of atherosclerosis [48]. However, as VCAM-1 assists in leukocyte adhesion to endothelium, it plays an important role in all stages of atherosclerosis [49].

Factors such as aging, smoking, and comorbidities worsen the development of atherosclerotic lesions, leading to challenges in accurately assessing the risk of cardiovascular disease. Varona et al. found that higher levels of sVCAM-1 were associated with an imbalanced pro-inflammatory state, suggesting the existence of subclinical atherosclerosis. These data could improve the assessment of cardiovascular disease risk in non-smoking patients with metabolic syndrome (MetS), exceeding traditional scoring methods. Patients with MetS had a higher prevalence of carotid plaque, carotid intima-media thickness above 0.9, and increased coronary calcium score compared to those without MetS. The research discovered a strong connection between these elements and a prominent increase in plasma sVCAM-1 levels [50].

Atherosclerotic cardiovascular complications are a major cause of mortality in patients who undergo hemodialysis. The correlation between Intima Media Thickness and sVCAM-1 was found to be weak [51].

Risk factors that contribute to the development of atherosclerosis and venous thromboembolism have similarities and are primarily linked to dysfunction of the endothelium. Results from the study of Dzikowska-Diduch et al. showed that individuals with low levels of E-selectin and high levels of sICAM-1 are at a greater risk of experiencing repeat occurrences of thromboembolism [52].

## 6. The Role of Cell Adhesion Molecules in Other Inflammatory Diseases

Schizophrenia, like atherosclerosis and psoriasis, is linked to ongoing inflammation at a low level, which has been correlated with increased chances of vascular issues and cardiovascular disease. Furthermore, it is characterized by a rise in cellular adhesion molecules that serve as markers for vascular endothelial activity.

Nguyen et al. showed that patients with schizophrenia who had more damage to their endothelium experienced the onset of symptoms at a younger age. These patients also had higher blood pressure, lower levels of high-density lipoprotein cholesterol, increased resistance to insulin, lower mental well-being, and a higher risk of developing coronary heart disease according to the Framingham scoring system [53].

Cell adhesion molecules are essential for determining where T cells are in the skin. Localization of T lymphocytes in the skin is vital for immune monitoring and in the development of skin conditions such as cutaneous T-cell lymphoma (CTCL). Lopez et al. focused on examining the concentrations of sVCAM-1 and sE-selectin in the blood of patients with CTCL. The study found that there was no notable increase in the levels of soluble E-selectin and sVCAM-1 [54].

Selectins are crucial for effector T cells to migrate to areas of inflammation, and blocking selectin activity has resulted in a decrease in the severity of certain diseases in animal experiments. In the mouse model of rheumatoid arthritis, the use of anti-VCAM-1 monoclonal antibodies at the beginning of collagen immunization was found to decrease the severity of the disease. However, this treatment did not exhibit the same effectiveness when administered after the disease had already developed. The main benefit of the treatment was observed in a reduction in the number of affected joints. Anti-VCAM-1 has also been demonstrated to improve bowel inflammation in a colitis mouse model [55].

The presence of adhesion receptors on endothelial cells is believed to play a significant role in the movement of cells into tissues. Skin is a specialized environment for the movement of white blood cells during an inflammatory response. Das et al. proved that there was a higher level of VCAM-1 expression on endothelial cells in the affected areas of contact dermatitis compared with samples taken from individuals with psoriasis [56].

## 7. Other Factors That May Influence VCAM-1 and E-Selectin Levels

### 7.1. Metabolic Syndrome and Hyperglycemia

Zemlin et al. investigated the correlation between levels of E-selectin, carotid intima media thickness (CIMT), and cardiovascular and metabolic characteristics in South Africans of mixed ancestry who have normal and high blood sugar levels. The levels of E-selectin were significantly elevated in the hyperglycemic group compared to the normoglycemic group. Significant variations were observed between the two groups in terms of their indicators for blood sugar levels and body fat, but not for CIMT. Strong associations were observed between E-selectin and age, indicators of blood sugar levels and inflammation, excessive fat in the abdominal area, and factors related to lipid levels. In robust linear regression models, the associations remained significant only with age, hyperglycemia, and C-reactive protein. CIMT, which was not related to E-selectin, was primarily influenced by age and gender in the regression models. The relationship between high blood sugar levels and the concentration of E-selectin may indicate damage to the endothelium [57].

In the study of Ghazizadeh et al., patients suffering from MetS and diabetes mellitus showed a noticeably elevated serum E-selectin concentration. Participants who had elevated levels of E-selectin in their blood also had higher levels of hs-CRP, FBG, TG, uric acid, BMI, and lower levels of HDL-C in their blood. There were notable connections found between the levels of E-selectin in the blood and both the occurrence of metabolic syndrome as well as its associated risk factors [58].

### 7.2. Sex and Age

The Stanislas study assumed the measurement of biological determinants of serum ICAM-1, E-selectin, P-selectin, and L-selectin levels in healthy subjects. The children, both boys and girls aged 4–17 years, showed a decrease in the levels of ICAM-1, E-selectin, P-selectin, and L-selectin. There was no difference observed between genders. In adults, there is no difference in the levels of ICAM-1, E-selectin, and P-selectin based on age. However, the level of L-selectin decreases until the age of 55. Additionally, there have been no observed variations in the levels of ICAM-1, E-selectin, and L-selectin between males and females. The level of E-selectin was found to be correlated with higher body mass index (BMI), leukocyte, platelet, and erythrocyte counts, glucose, alkaline phosphatase (ALP), and TNF-alpha. On the other hand, it was negatively associated with the use of oral contraceptives (OC) [59].

### 7.3. Migraine and Tobacco Smoking

Michalak et al. emphasized that migraineurs who smoke tobacco exhibit higher levels of E-selectin in their bloodstream. In individuals with migraines who smoke, the processes that control E-selectin levels are disrupted, making them more vulnerable to a decrease in E-selectin levels during high blood sugar levels. The protective impact of HDL cholesterol on the rise in E-selectin levels is not significant. Authors demonstrated that individuals who smoke tobacco and experience migraines have decreased levels of homocysteine. In comparison to the control group, smoking migraine patients showed higher levels of E-selectin. Additionally, there was an inverse relationship between levels of E-selectin and glycemia in individuals with migraines who smoke. The disruption of the positive impact of HDL cholesterol, which defends against raised levels of E-selectin, was observed [60].

Wang et al. showed that atorvastatin may partially counteract the endothelial inflammation caused by tobacco smoking by blocking the NF-κB signal pathway. The study findings indicated that the levels of VCAM-1, E-selectin, and NF-κB were notably elevated in the cigarette smoking extract (CSE) group compared to the other two groups (control group and atorvastatin + CSE group). The researchers discovered that the levels of VCAM-1, E-selectin, and NF-κB were slightly increased in the group treated with CSE and atorvastatin compared to the control group [61].

## 8. Conclusions

In conclusion, the complex relationship between VCAM-1 and E-selectin in inflammatory diseases, particularly in the context of psoriasis, highlights their crucial functions in controlling leukocyte recruitment, activating endothelial cells, and disease pathogenesis. The findings presented emphasize the potential of VCAM-1 and E-selectin as important biomarkers for cardiovascular risk in psoriasis, shedding light on their implications in atherosclerosis, vasculitis, cancer, and other inflammatory conditions.

The exploration of therapeutic interventions, including TNF-α inhibitors and biological therapies that target selectins, reveals encouraging possibilities for enhancing endothelial function, reducing inflammation, and achieving better health outcomes in patients with psoriasis. Additionally, the influence of systemic treatments and UVB phototherapy on VCAM-1 and E-selectin levels underscores their significance as markers for treatment effectiveness and the severity of the disease.

Moreover, the impact of various factors such as metabolic syndrome, high blood sugar levels, age, sex, migraines, and tobacco smoking on VCAM-1 and E-selectin levels provides a comprehensive understanding of the diverse factors influencing endothelial activation and disease progression in inflammatory conditions. Overall, the findings presented in this review (summarized in Table 1) highlight the important functions of VCAM-1 and E-selectin as significant indicators for assessing inflammation, disease severity, and cardiovascular risk in patients with psoriasis. The common pathways shared between psoriasis and atherosclerosis, along with the potential of endothelial activation markers in guiding clinical management approaches, offer valuable insights for advancing research and improving outcomes in inflammatory diseases.

## Figures and Tables

**Table 1:** Summary and description of factors and co-morbidities that may affect adhesion molecule levels.

Factors/Comorbidities	Influence on Adhesion Molecules
Atherosclerosis	Blood levels of sE-selectin are noticeably higher in patients with psoriasis and can be used to assess disease activity. A strong correlation has been found between sE-selectin values and PASI scores. The same correlations were shown in individuals with atopic dermatitis and bullous dermatoses [18,27,29,30]. Increased CEC levels observed in patients with psoriasis may indicate endothelial damage. The decrease in the number of CECs after Etanercept treatment suggests that effective psoriasis treatment could potentially improve endothelial cell function. The study showed a significant difference in CEC levels between psoriasis patients and controls. This number had an inverse correlation with sE-selectin levels. After undergoing treatment for six months, patients showed a significant reduction in CEC levels and PASI score [31]. In young adults, there is a positive correlation between the levels of E-selectin in the bloodstream and the thickness of the aortic wall and pulse wave velocity. This suggests that these cell adhesion molecules may cause inflammatory changes in the arterial walls, leading to a higher risk of atherogenic diseases even at a young age [9]. sVCAM-1 seems to exhibit higher specificity in identifying atherosclerosis when compared to other markers. The concentration of sVCAM-1 in the serum seems to be associated with the severity of atherosclerosis and could potentially be used to identify early stages of atherosclerosis [10,13]. Individuals with low levels of E-selectin and high levels of sICAM-1 are at a greater risk of experiencing repeat occurrences of thromboembolism [16].
Phototherapy	UVA and UVB treatment reduce the presence of ICAM-1 and VCAM-1. UVB treatment interferes with the ability of endothelial cells to adhere to each other and selectively controls the expression of adhesion molecules in these cells. The efficacy of NB-UVB phototherapy was confirmed, as the treatment led to a significant reduction in PASI scores. Serum sE-selectin levels also decreased significantly after treatment. The efficacy of NB-UVB phototherapy in the treatment of psoriatic lesions may be due to a reduction in serum E-selectin [34,35,36,37].
Fumarates treatment	Inhibition of VCAM-1 and E-selectin expression on two different groups of endothelial cells was observed when dimethyl fumarate was used, with the reduction becoming stronger as its concentration increased. These findings indicate that endothelial cells experience significant functional changes in response to dimethyl fumarate, together with its already established effects on leukocytes. The effects of monoethyl fumarate and fumaric acid were not observed [38,40].
Methotrexate treatment	MTX therapy reduces E-selectin and VCAM-1 levels [41,44].
Biological treatment	TNFα may potentially have a significant effect on the activation of vascular adhesion molecules in psoriatic lesions. Terajima et al. examined the expression of VCAM-1 and E-selectin by immunohistochemistry in psoriatic skin and compared their expression levels in normal skin. Dermal dendritic cells and fibroblasts showed significant staining for VCAM-1, while endothelial cells from lesional skin showed significant staining for E-selectin [47,51]. Patients with psoriasis showed a decrease in E-selectin levels after treatment with infliximab. In addition, strong associations were observed between PASI measurements and E-selectin production [45,48]. Patients with moderate to severe psoriasis have been shown to exhibit endothelial activation, which is associated with features of the metabolic syndrome. Treatment with adalimumab reduced sE-selectin levels, indicating that anti-TNF-α therapy has a positive effect on processes associated with atherosclerosis progression in patients with psoriasis [32,49]. In addition, adalimumab causes a reduction in NVRI in psoriasis patients [46].
Inflammatory diseases	Schizophrenia is linked to ongoing inflammation at a low level, which has been correlated with increased chances of vascular issues and cardiovascular disease. Furthermore, it is characterized by a rise in cellular adhesion molecules that serve as markers for vascular endothelial activity [17].
Metabolic syndrome	A significant association was found between E-selectin levels and the presence of metabolic syndrome. E-selectin levels were significantly elevated in the hyperglycaemic patients [52,53].
Sex and age	Children, both boys and girls aged 4 to 17 years, showed a decrease in ICAM-1, E-selectin, P-selectin, and L-selectin levels. No difference was observed between the sexes. In adults, there is no difference in the levels of these molecules according to age and sex. However, L-selectin levels decline by the age of 55 [54].
Migraine and tobacco smoking	VCAM-1 and E-selectin levels are significantly elevated in tobacco smokers [56]. Compared to the control group, migraine patients who smoked tobacco showed higher levels of E-selectin [55].

Abbreviations: sE-selectin—soluble E-selectin; PASI—Psoriasis Area Severity Index; CEC—circulating endothelial cell; sVCAM-1—soluble vascular cell adhesion molecule—1; sICAM-1—soluble intercellular adhesion molecule—1; ICAM-1—intercellular adhesion molecule—1; VCAM-1—vascular cell adhesion molecule—1; NB-UVB—narrow-band ultraviolet B; MTX—methotrexate; TNFα—tumor necrosis factor α; NVRI—nailfold vessel resistance index.

## Data Availability

Data sharing not applicable.
